# Microwave-Induced Synthesis of Schiff Bases of Aminothiazolyl Bromocoumarins as Antibacterials

**DOI:** 10.4103/0250-474X.40338

**Published:** 2008

**Authors:** K. N. Venugopala, B. S. Jayashree

**Affiliations:** Department of Pharmaceutical Chemistry, Al-Ameen College of Pharmacy, Near Lalbagh Main Gate, Hosur Road, Bangalore - 560 027, India; 1 Department of Pharmaceutical Chemistry, Manipal College of Pharmaceutical Sciences, MAHE, Manipal - 576 104, India

**Keywords:** Bromocoumarin, microwave, characterization, antibacterials

## Abstract

A fast and highly efficient method for the synthesis of some of the schiff bases of aminothiazolylbromocoumarin (4a-m) has been performed by microwave irradiation of 2′-amino-4′-(6-bromo-3-coumarinyl) thiazole (3) and substituted aromatic aldehydes (a-m). Microwave assisted reactions have resulted in better yields of the desired products than when prepared under conventional conditions. The resulting products were evaluated for their qualitative and quantitative antibacterial activity.

The synthesis of coumarins and their derivatives has attracted the attention of organic and medicinal chemists as these are widely used as fragrances, pharmaceuticals and agrochemicals^1^. Benz-*2*pyrones and its heterocyclic derivatives, in particular schiff bases and carboxamides of 3-thiazolyl substituted coumarins, display important biological properties such as analgesic, anti-inflammatory^2^,^3^, anticoagulant^4^, antimicrobial, antiviral^5^ and HIV protease inhibitory^6^ activities. Potent antibiotics like novobiocin, coumaromycin and charteusin are coumarin derivatives. Consequently, we were involved in the synthesis and chemistry of schiff bases and carboxamides of aminothiazolyl substituted coumarins. As a continuation of our research in this area, the present work was aimed at the synthesis of schiff bases of 2-amino thiazolyl bromocoumarin by microwave-assisted method. Microwave irradiation has become a very useful tool in organic synthesis and has been explored extensively since the last decade. Microwave irradiation often leads to a remarkable decrease in reaction time, increased yields and easier workup matching with green chemistry protocols. The resulting compounds of Scheme 1 were characterized by ^1^H-NMR and mass spectral studies. X-ray study was made on parent compound (3) and the test compounds were subjected to qualitative and quantitative antibacterial activity by cup plate method and ELISA technique, respectively.

**Scheme 1:**
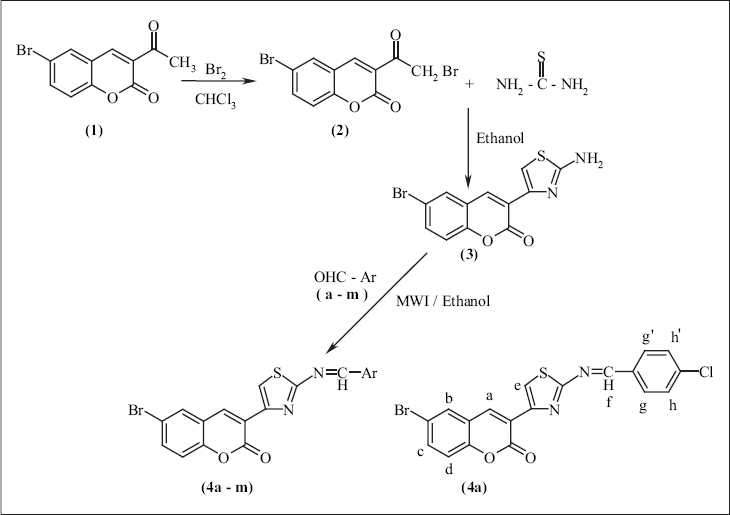
Synthesis of compounds (4a-m). Where Ar: a = 4-Cl C_6_H_4_, b = 3,4,5-OCH_3_ C_6_H_2_, c = 2-NO_2_ C_6_H_4_, d = 3-NO_2_ C_6_H_4_, e = 4-OH, 3-OCH_3_ C_6_H_3_, f = 2-OH, 5-Br C_6_H_3_, g = 4-N(CH_3_)_2_ C_6_H_4_, h = 2-CH_3_ C_6_H_4_, i = 2-OH C_6_H_4_, j = 2-OCH_3_ C_6_H_4_, k = C_6_H_5_, l = 3,4-OCH_3_ C_6_H_3_ and m = 4-NO_2_ C_6_H_4_.

Melting points were determined in open capillaries and are found uncorrected. IR spectra were recorded on Fourier transform IR spectrophotometer Model Shimadzu 8700 using KBr disc method. ^1^H-NMR spectra were recorded on AMX-400 liquid state NMR spectrometer in CDCl_3_ using tetramethylsilane as an internal standard. Mass spectra were recorded on Jeol JMS DX303 Mass spectrometer with Electron Impact Ionization (EII). The purity of the products was determined by thin layer chromatography using several solvent systems of different polarity. The compounds were analyzed for C, H and N and the values were found within ±0.4% of the calculated values. The microwave oven used was conventional kitchen microwave oven. The yield and reaction time of the products are reported in Table 1.

**TABLE 1:** COMPARISON OF REACTION TIME AND YIELDS OF THE TEST COMPOUNDS (4a-m)

Comp. No.	Yield (%)			Reaction period (min)		
		
	Method A (conven)	Method B (conven)	Method C (MORE^a^)	Method A (min)	Method B (min)	Method C (sec)
4a	62	57	88	120	120	105
4b	71	60	89	90	90	66
4c	58	53	73	120	120	110
4d	60	55	77	120	120	110
4e	66	64	82	150	150	108
4f	66	64	83	90	90	70
4g	69	63	89	120	120	100
4h	64	62	80	120	120	103
4i	68	64	87	60	60	65
4j	62	60	89	120	120	100
4k	78	74	91	90	90	68
4l	67	63	85	120	120	113
4m	64	61	81	120	120	106

a Isolated yields

The synthesis of 2′-amino-4′-(6-bromo-3-coumarinyl) thiazole^2^ (3) was achieved by cyclization of 3-bromoacetyl-6-bromocoumarin (2) with thiourea in absolute ethanol medium in the presence of piperidine as catalyst and the resulting compounds (4a-m) were obtained by microwave irradiation of compound (3) and different aromatic aldehydes (a-m) in absolute ethanol with different time intervals. The synthetic route is shown in Scheme 1.

In conventional refluxing method (method A), compound (3) (0.01 mol) and substituted aromatic aldehydes (a-m) (0.01 mol) were taken in absolute alcohol (20 ml) and refluxed for 2 h, cooled and poured into crushed ice. The precipitate obtained was recrystallized using aqueous dimethyl sulfoxide and ethanol.

In conventional heating method (method B), compound (3) (0.01 mol) and substituted aromatic aldehydes (a-m) (0.01 mol) were taken in a round bottom flask and heated on an oil bath at 180°, cooled and the melted reaction medium was reprecipitated with aqueous ethanol and recrystallized using dimethyl sulfoxide and ethanol.

As in microwave-induced organic reaction enhancement (MORE, Method C), compound (3) (0.01 mol) and substituted aromatic aldehydes (a-m, 0.01 mol) in ethanol (30 ml) were taken into a 250 ml conical flask and capped with a glass funnel and subjected to microwave irradiation for 65-113 seconds at an interval of every 20 seconds at 260 watts. On completion of the reaction, followed by TLC examination, the mixture was cooled to room temperature and the product was poured into crushed ice. The crude products (4a-m) were purified by recrystallization from ethanol and dimethyl sulfoxide. The characterization data of the synthesized test compounds (4a-m) are tabulated in Table 2.

**TABLE 2:** CHARACTERIZATION DATA OF THE SYNTHESIZED TEST COMPOUNDS (4a-m)

Comp. No.	m.p (°)		Recrystalizing solvent	% Required (found)			IR (cm^−1^v)
		
	Found^b^	Required		C	H	N	
3	210-212	211	ethanol	44.60(44.61)	2.18(2.16)	8.67(8.66)	1720
4a	254-256	255	aq. DMSO	55.49(55.48)	2.70(2.62)	6.81(6.70)	1735
4b	234-236	234	aq. DMSO	52.71(52.69)	3.42(3.30)	5.59(5.50)	1726
4c	242-244	243	aq. DMSO	50.02(49.96)	2.21(2.10)	9.21(9.20)	1722
4d	254-256	256	aq. DMSO	50.02(49.90)	2.21(2.16)	9.21(9.18)	1733
4e	234-236	235	aq. DMSO	52.53(52.41)	2.87(2.70)	6.13(6.05)	1719
4f	274-276	276	aq. DMSO	45.09(44.93)	1.99(1.82)	5.53(5.50)	1730
4a	180-182	180	aq. DMSO	55.52(55.50)	3.55(3.44)	9.25(9.16)	1728
4h	218-220	218	aq. DMSO	56.48(56.46)	3.08(2.90)	6.59(6.47)	1725
4i	222-224	224	ethanol	53.41(53.30)	2.59(2.52)	6.56(6.49)	1732
4j	148-150	150	aq. DMSO	54.43(54.39)	2.97(2.81)	6.35(6.24)	1731
4k	224-226	225	aq. DMSO	55.49(55.60)	2.70(2.62)	6.81(6.72)	1733
4l	212-214	214	aq. DMSO	53.52(53.41)	3.21(3.11)	5.94(5.85)	1730
4m	264-266	264	aq. DMSO	50.02(49.66)	2.21(2.14)	9.21(9.18)	1735

^a^ll the test compounds were characterized by IR spectral analysis and by comparison of their physical properties with those of the authentic compounds^3^.

b Melting points of the compounds are consistent with reported values.

Compound 4a: IR (KBr, cm^−1^v) 3042, 1735 (lactone-C = O), 1676, 1606, 1548, 1355, 1231, 835, 769, 744, 558. ^1^H-NMR: (400 MHz, CDCl_3_) 8.98 (s, 1H, -N = CH-), 8.73 (s, 1H, Hetero Ar-H), 8.42 (s, 1H, Hetero Ar-H), 7.97 (d, 2H, Ar-H), 7.75 (d, 1H, Ar-H), 7.65 (dd, 1H, Ar-H), 7.51 (d, 2H, Ar-H), 7.27 (d, 1H, Ar H). MS: m/z 445 (M^+^ 100), 416 (10), 390 (5), 366 (6), 339 (5), 321 (10), 280 (12), 250 (35), 220 (22), 196 (76), 182 (16), 165 (7), 145 (53), 129 (12), 97 (25), 83 (32), 69 (41), 57 (57), 43 (46).

X-ray powder diffraction pattern was recorded on the parent compound (3) in STOE powder diffractometer using Debye-Scherrer Geometry (Indian Institute of Science, Bangalore) wave length CuKα(λ = 1.54178 Å. Cell parameters A = 13.874 (0.006) Å, B = 7.054 (0.002) Å, C = 12.505 (0.007) Å, α = β = γ = 90.0°. Crystal system was orthorhombic.

Antibacterial screening of the synthesized compounds was carried out by cup-plate method^7^ using two strains i.e., *Bacillus subtilis* (ATCC 6633) and *Escherichia coli* (ATCC 8739). Ampicillin was used as reference sample and antibacterial activity of the test compounds (4a-m) is presented in Table 3. The minimum inhibitory concentration of the test compounds showing promising activity was determined using 96-well plate (two fold dilution technique) and an ELISA Reader^8^.

**TABLE 3:** THE ANTIBACTERIAL ACTIVITY OF THE TEST COMPOUNDS (4a-m)

COMP. No.	Cup plate method		MIC (μg)	
		
	*B. subtilis*	*E. coli*	*B. subtilis*	*E. coli*
3	++	++	185.00	197.00
4a	+++	+++	147.00	141.00
4b	+	+	241.00	239.00
4c	++	++	195.00	183.00
4d	++	++	180.00	177.00
4e	+	+	225.00	220.00
4f	+	+	247.00	255.00
4a	+	+	280.00	283.00
4h	+	+	265.00	247.00
4i	++	++	192.00	201.00
4j	+	+	216.00	210.00
4k	+	+	260.00	265.00
4l	++	++	190.00	176.00
4m	++	++	178.00	180.00
Ampicillin	++++	++++	145.00	135.00

+ : Less active (0.2-0.5 mm);

++ : Moderately active (0.6-1.4 mm);

+++ : Highly active (1.5-3.0 mm);

++++ : Very highly active (over 3.00 mm)

Structure of the synthesized schiff bases was supported by IR, ^1^H-NMR and Mass spectral studies. In IR spectra, a prominent peak was observed for lactone of coumarins (1), (2), (3) and (4a-m) from 1735-1719 cm^−1^v. In ^1^H-NMR spectra, the signal due to −N=CH- protons appeared as singlet at 8.98, heteroAr-H(d) proton appeared as singlet at 8.73, heteroAr-H(e) proton appeared as doublet at 8.42, Ar-H(g,g') two protons appeared as doublet at 7.97 (J = 8.27cps), Ar-H(c) proton appeared as doublet at 7.75, Ar-H(b) proton appeared as doublet of doublet at 7.65, Ar-H(h,h') proton appeared as doublet of doublet at 7.51 and Ar-H(a) proton appeared as doublet at 7.27. Molecular ion peak was observed at 445 and base peak at 196. These observations supported the formation of the resulting compound (4a). Out of the fourteen compounds subjected for qualitative antibacterial activity, one of the test compounds (4a), was shown to be active greater than that of test compounds such as (4), (4c), (4d), (4i), (4l) and (4m). All the test compounds were subjected for quantitative antibacterial determination and compounds, such as (4a), showed minimum inhibitory concentration at 147 μg and 141 μg against *Bacillus subtilis* and *Escherichia coli,* respectively when compared to that of the activity against standard drug ampicillin.
